# A New Denoising Method for UHF PD Signals Using Adaptive VMD and SSA-Based Shrinkage Method

**DOI:** 10.3390/s19071594

**Published:** 2019-04-02

**Authors:** Jun Zhang, Junjia He, Jiachuan Long, Min Yao, Wei Zhou

**Affiliations:** 1 State Key Laboratory of Advanced Electromagnetic Engineering and Technology, Huazhong University of Science and Technology, Wuhan 430074, China; zhangjunwh33@sina.com (J.Z.); junjiahe_wh@sina.com (J.H.); 2 School of Electronics and Information Engineering, Wuhan Donghu University, Wuhan 430212, China; minyao@whu.edu.cn; 3 Metrology Department, China Electric Power Research Institute, Wuhan 430074, China; zhouweiwish@163.com

**Keywords:** UHF PD signals, denoising, adaptive variational mode decomposition, singular spectrum analysis, threshold shrinkage

## Abstract

Noise suppression is one of the key issues for the partial discharge (PD) ultra-high frequency (UHF) method to detect and diagnose the insulation defect of high voltage electrical equipment. However, most existing denoising algorithms are unable to reduce various noises simultaneously. Meanwhile, these methods pay little attention to the feature preservation. To solve this problem, a new denoising method for UHF PD signals is proposed. Firstly, an automatic selection method of mode number for the variational mode decomposition (VMD) is designed to decompose the original signal into a series of band limited intrinsic mode functions (BLIMFs). Then, a kurtosis-based judgement rule is employed to select the effective BLIMFs (eBLIMFs). Next, a singular spectrum analysis (SSA)-based thresholding technique is presented to suppress the residual white noise in each eBLIMF, and the final denoised signal is synthesized by these denoised eBLIMFs. To verify the performance of our method, UHF PD data are collected from the computer simulation, laboratory experiment and a field test, respectively. Particularly, two new evaluation indices are designed for the laboratorial and field data, which consider both the noise suppression and feature preservation. The effectiveness of the proposed approach and its superiority over some traditional methods is demonstrated through these case studies.

## 1. Introduction

Power systems constitute critical societal infrastructure, and play an increasingly important role in the development of the national economy and the improvement of the population’s living standard [1]. Accompanying inevitable technological advances, the probability of failure of electrical equipment and the resulting damage will also increase greatly. Therefore, timely and accurate monitoring of the operating status of electrical equipment plays a vital role to ensure its safe and stable operation, and to prevent large-scale blackouts. In most cases, high-voltage electrical equipment is accompanied by partial discharge (PD) phenomenon before catastrophic failure [2,3,4]. Among various PD detection methods, the ultra-high frequency (UHF) technique has been widely used due to its superior performance in location [5], type recognition [6] and severity evaluation of PD faults [7]. However, the complicated and unpredictable electromagnetic environment in a substation may cause the PD signals to be contaminated by noise, leading to an incorrect diagnosis. Hence, a reliable noise suppression method is a prerequisite for the accurate detection and diagnosis of PD [8].

Up to now, various methods for PD signal denoising have been reported, most of which are designed to deal with a particular noise type (e.g., white noise or narrow band noise). To our best knowledge, most state of the art PD denoising methods are based on signal decomposition technologies, such as the wavelet transform (WT), empirical mode decomposition (EMD), singular value decomposition (SVD) and sparse decomposition, etc. For example, the WT-based denoising methods was studied comprehensively in [9]. Recently, the dual-tree complex WT was introduced for PD denoising, and it shows superior performance in comparing with traditional WT and SVD [10]. Although the WT-based method has been widely accepted as a promising tool for denoising, the performance of WT relies heavily on the selection of wavelet basis, decomposition levels and threshold, especially when the composition of PD signal is complex or the PD component is relatively weak. An improper selection of these parameters may lead to the loss of some effective components. Different from WT, EMD and SVD are non-parametric signal analysis tools which can be implemented without pre-defined basis functions. In [11], a discrete spectral interference suppressing method was developed based on bivariate EMD, and it showed superior performance over WT in PD denoising. Authors in [12] also introduced the EMD algorithm into ultrasonic PD signals, and the denoising result was satisfied. Besides, some improved version of EMD like ensemble EMD (EEMD) [13] or complete ensemble EMD (CEEMD) [14] also perform well in noise suppression. However, all these EMD-based methods are essentially a kind of recursive algorithm, which suffers the following deficiencies: (i) the previous estimation error will be passed to the following results, namely error accumulation; (ii) mode mixing will exist when dealing with multi-components and strong nonlinear signals; (iii) there are end effects in this kind of algorithms. Another attractive decomposition method is SVD. An adaptive SVD method was proposed in [15], and its key innovation was that it can select and remove those singular values (SVs) relating to white noise automatically. To improve the efficiency of traditional SVD, a sliding SVD was presented in [16]. It provides a fully automatic data-driven component extraction scheme and a sliding window-based SVD (just like the Short Time Fourier Transform). Nevertheless, because the selected SVs are still mixed with small amount of white noise, using SVD alone may result in unsatisfactory denoising results. In addition, when the original signal contains multiple components, it will become difficult for SVD to distinguish other components except the white noise. Another new denoising method based on sparse decomposition was proposed by designing PD-correlated atom and overcomplete dictionary [17], and superior results could be achieved over traditional WT methods in noise suppression and waveform distortion.

Recently, a completely non-recursive mode decomposition method, namely the variational mode decomposition (VMD) was developed [18]. With this method, the original signal can be decomposed into a set of amplitude modulation-frequency modulation functions (AM-FM), which are band-limited and frequency-unmixed. Due to its superior performance over traditional methods like WT and EMD, researchers in various fields such as machinery [19], biomedicine [20], geology [21] and so on, have begun to apply VMD extensively. However, the original author also pointed out that the performance of VMD will be affected by the following three parameters: the number of modes *K*, the quadratic penalty term *a* and the time-step of the dual ascent *τ* [18]. Some guidelines for choosing these parameters were discussed in [18], for example, *τ* is suggested to be zero when the noise level is high. Moreover, a relatively moderate value of *a* recommended by the author is 2000. Thus, the determination of *K* is the key problem when employing VMD.

This paper aims to remove various interferences in UHF PD signal and preserve its key features at the same time. To achieve this goal, a new denoising method, namely the adaptive variational mode decomposition and singular spectrum analysis (AVMDSSA), is developed, and the main steps are outlined as follows:

(i) An automatic VMD algorithm is presented based on a mode-mixing judgement criterion. With the optimal *K*, the original PD signal can be decomposed into BLIMFs at high accuracy.
(ii) Considering that BLIMFs containing PD components will exhibit the shape of pulse, a kurtosis-based method is employed to pick out those valuable BLIMFs (i.e., eBLIMFs).
(iii) For each selected eBLIMF, the dominant singular values (DSVs) are retained at first. Then, they will be used to reconstruct PD signal by diagonal averaging. Next, the rescaling thresholding technique [9] is applied to further remove the residual white noise in each eBLIMF. Finally, the denoised UHF PD signal is obtained by adding up all these denoised eBLIMFs.

The rest of this paper is organized as follows: Section 2 reviews the mathematical background. The proposed method is detailed in Section 3. Simulative case, laboratorial case and field case are respectively analyzed in Section 4, Section 5 and Section 6. Main contributions and some open questions of our paper is discussed in Section 7. Finally, the conclusion is drawn in Section 8.

## 2. Mathematical Background

### 2.1. Variational Mode Decomposition

In VMD, the principle modes of a signal are redefined as a set of AM-FM functions, which can be expressed as:

(1) $$u_{k}(t)=A_{k}(t)\cos(\phi_{k}(t))$$

where $A_{k}(t)$ and the derivative of $\phi_{k}(t)$ are non-negative. The VMD algorithm mainly includes the following two steps:

(i) Construction of the variational problem

The purpose of this step is to obtain *K* mode functions (i.e., $u_{k}(t)$ in Equation (1)) that minimize the summation of the bandwidths of all modes, under the constraint that the sum of all modes is equal to the original signal. This problem can be formulated as the following constrained variational problem:

(2) $$\underset{\left\{u_{k}\},\{w_{k}\right\}}{\min}\left\{\sum_{k=1}^{K}{\left\|\partial_{t}\left[\left(\delta (t)+\frac{j}{\pi t}\right)*u_{k}(t)\right]e^{-jw_{k}t}\right\|}_{2}^{2}\right\},\;\;\;\;\;\;s.t.\;\;\;\sum_{k=1}^{K}u_{k}=f$$

where $u_{k}$ is the *k*th BLIMF, and $w_{k}$ is the center frequency of $u_{k}$. Notation ${\left\|\right\|}_{2}^{2}$ denotes the squared *L*^2^ norm, $\partial_{t}$ is derivative operator, and δ(t) is the Dirac function.

(ii) Solving the above variational problem

To solve the Equation (2), the constrained variational problem should be transformed into unconstrained first. This can be achieved by introducing the Lagrangian multiplier λ and quadratic penalty term α. The new unconstrained problem can be formulated as:

(3) $$\begin{array}{ll} L(\{u_{k}\},\{w_{k}\},\lambda ): & =\alpha \sum_{k=1}^{K}{\left\|\partial_{t}\left[\left(\delta (t)+\frac{j}{\pi t}\right)*u_{k}(t)\right]e^{-jw_{k}t}\right\|}_{2}^{2}+{\left\|f(t)-\sum_{k=1}^{K}u_{k}(t)\right\|}_{2}^{2}\\ & +\langle \lambda (t)\;,\;\;f(t)-\sum_{k=1}^{K}u_{k}(t)\rangle \end{array}$$

Through a series of mathematical derivations based on the Alternate Direction Method of Multipliers (ADMM) and Parseval/Plancherel Fourier isometry, the above problem can be solved in the spectral domain as follows:

(4) $${\hat{u}}_{k}^{n+1}(w)=\frac{\hat{f}(w)-\sum_{i\neq k}^{}{\hat{u}}_{i}(w)+\frac{\hat{\lambda }(w)}{2}}{1+2\alpha {\left(w-w_{k}\right)}^{2}}$$

(5) $$w_{k}^{n+1}=\frac{\int_{0}^{\infty }w{\left|{\hat{u}}_{k}(w)\right|}^{2}dw}{\int_{0}^{\infty }{\left|{\hat{u}}_{k}(w)\right|}^{2}dw}$$

Based on the above, the complete process of the VMD method is summarized as follows:

Step 1: Initialize the parameters of the first loop $\left\{u_{k}^{1}\right\},\;\left\{w_{k}^{1}\right\},\;\lambda^{1}$, k=1,2,…,K, and K is the predefined number of decomposed modes. In addition, set the cycle index n=0;
Step 2: Let n=n+1, then begin the outer loop;
Step 3: Execute the first inner loop according to Equation (4) to update the K BLIMFs in the spectral domain $\left\{{\hat{u}}_{k}^{n+1}(w)\right\}$;
Step 4: Execute the second inner loop according to Equation (5) to update the center frequencies of all BLIMFs in the spectral domain $\left\{w_{k}^{n+1}\right\}$.
Step 5: Update the Lagrangian multiplier by the following expression:
  (6) $${\hat{\lambda }}^{n+1}(w)={\hat{\lambda }}^{n}(w)+\tau \left(\hat{f}(w)-\sum_{k}{\hat{u}}_{k}^{n+1}(w)\right)$$
Step 6: Repeat the algorithm from Step 2 to Step 5 until the following condition is satisfied:
  (7) $$\sum_{k}^{}{\left\|{\hat{u}}_{k}^{n+1}-{\hat{u}}_{k}^{n}\right\|}_{2}^{2}/{\left\|{\hat{u}}_{k}^{n}\right\|}_{2}^{2}<\varepsilon$$

### 2.2. Singular Spectrum Analysis

For a discrete finite-duration signal $X=\{x_{n},n=1...N\}$, SSA is usually applied to decompose X into a set of physically interpretable components. These components may contain different features of the X, thus SSA is considered to be a power tool for signal denoising or classification [22]. The main process of SSA can be summarized as follows:

Step 1: Embedding

In this step, the original signal X will be mapped into a trajectory matrix. Many matrix forms can be adopted to build a trajectory matrix, such as Toeplitz matrix, cycle matrix, or Hankel matrix. Among them, the Hankel matrix is most widely used due to its zero-phase shift property [23]. It is made up of *M* column vectors of length *L*, as shown in Equation (8).

(8) $$A=\left[\begin{array}{cccc} x_{1} & x_{2} & \cdots & x_{M}\\ x_{2} & x_{3} & \cdots & x_{M+1}\\ \vdots & \vdots & \vdots & \vdots \\ x_{L} & x_{L+1} & \cdots & x_{N} \end{array}\right]$$

where M=N−L+1. One of the important features of Hankel matrix is that it has equal diagonal elements. In addition, it should be noted that the parameter L will have great impact on the results of SSA, thus it should be carefully selected for specific application. 

Step 2: Decomposition

By using SVD, the Hankel matrix will be decomposed into a sum of sub-space matrices, which are orthogonal with each other. This process can be formulated as:

(9) $$A_{L\times M}=U_{L\times L}\sum_{L\times M}V_{M\times M}=\sum_{i=1}^{R}A_{i},\;\;\;A_{i}=\sigma_{i}u_{i}v_{i}^{T}$$

where $\sum =\mathrm{diag}(\sigma_{1},\ldots ,\sigma_{i},\ldots \sigma_{R})$, R=min(L,M) is the rank of the Hankel matrix, and $\sigma_{i}$ is the *i*th SV in descending order. In addition $u_{i}$ and $v_{i}$ are the column vectors of orthogonal matrices U and V, respectively. 

Step 3: Grouping

This step aims at dividing the matrices $\left\{A_{1},A_{2},\ldots ,A_{R}\right\}$ into r disjoint groups $I_{m}\;(m=1,\ldots ,r)$ according to the characteristics of the sub-components within the raw signal. Therefore, summation of the matrices in $I_{m}$ can be denoted as $A_{I_{m}}=\sum_{i\in I_{m}}A_{i}$. Adding all $A_{I_{m}}$ together will reconstruct the original Hankel matrix A. 

Step 4: Diagonal averaging

A common way to recover the signal X is called the diagonal averaging method, which uses the average of the diagonal elements of reconstructed Hankel matrix as the element of X. 

## 3. Proposed Denoising Method

### 3.1. Adaptive VMD

As discussed before, the performance of VMD is heavily dependent on the choice of its parameters, especially for the number of modes K. Some related studies have discussed the parameter selection problem of VMD, which mainly focus on optimizing by heuristic optimization algorithms. For example, a searching method for quadratic penalty term α and mode number K is presented in [24] based on the artificial fish swarm (AFS) algorithm. Besides, the particle swarm optimization (PSO) algorithm has also been applied to VMD in [8]. Although these methods can obtain appropriate parameters to some extent, they may need to go through many iterations before convergence. Moreover, one may have to set initial parameters of heuristic optimization algorithms manually, making the VMD method more complicated. 

**Figure 1:**
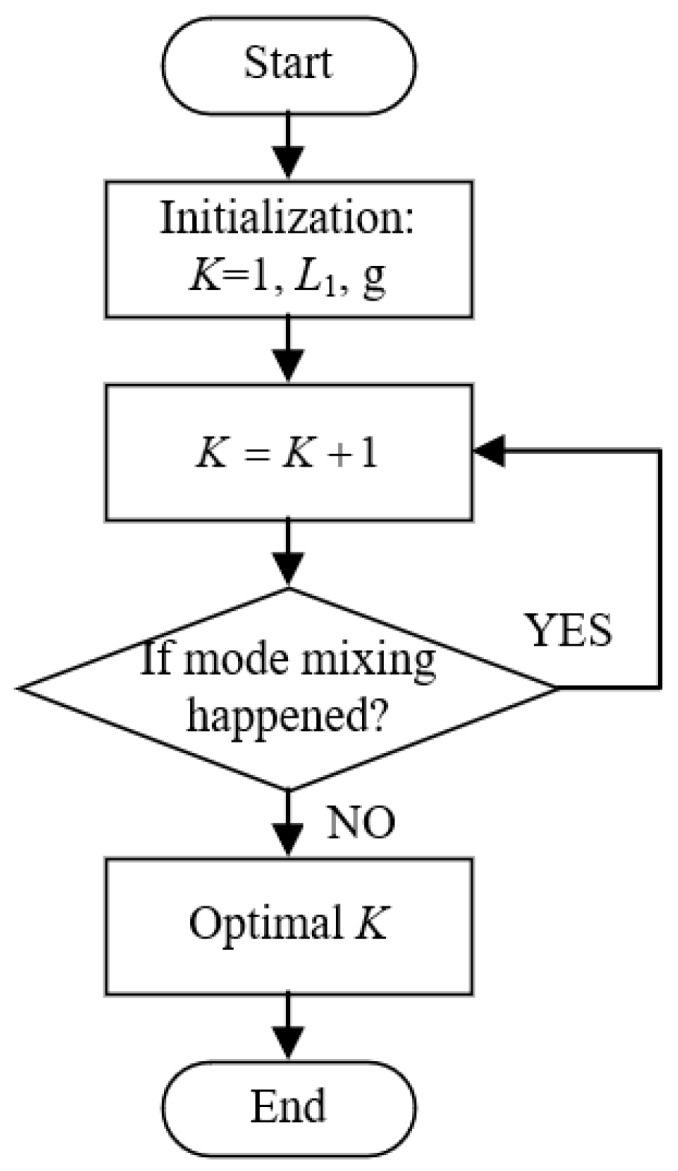
Flow chart of the proposed AVMD.

In this paper, the quadratic penalty term *a* and time-step of the dual ascent *τ* are respectively set to 2000 and 0, and we focus on the optimization method of the mode number *K*. The basic idea behind this adaptive VMD (AVMD) method is simple and straight: increase the value of *K* (starting from 2) step by step and obtain the BLIMFs by VMD during each step. When there is no mode mixing happened in all BLIMFs for a certain *K*, then this value is considered as optimal. Based on this concept, the schematic diagram of the proposed method is demonstrated in Figure 1. 

As can be seen from Figure 1, the key of this method is to determine whether there is mode mixing occurred in decomposed BLIMFs. For this purpose, the first important task is to find out the local maximum points (LMPs) of BLIMF spectrum, which are considered to belong to the potential effective components. This can be achieved by the following two steps: 

(i) Preliminary screening

To begin with, the frequency spectrum is divided into several consecutive segments, and each segment has the same length *L_1_* (the last segment may not). Then, the maximal point in each segment will be picked out to form a new sequence $segmax=\left\{seg\_1_{max},\ldots ,seg\_n_{max},\ldots ,\;seg\_{\tilde{N}}_{max}\right\}$, where $seg\_n_{max}$ denotes the maximal point of the *n*th segment, and ${\tilde{N}}_{max}$ is the number of segments.

(ii) Peaks confirmation

After the previous process, it is easy to find that the LMPs of the frequency spectrum must be the points within *segmax*. Therefore, the LMPs can be obtained easily by searching the peak points of *segmax*, remarked as *pks*.

Due to the influences of potential multiple components or interfering noise, the number of *pks* is likely to be more than one. Consequently, the remaining problem is to determine whether other points in *pks* except for the maximum are active components. Denote the maximal point of *pks* as *max_temp*, then the proposed rules for mode-mixing determination is given as follows: for any point in *pks* except for *max_temp*, if the distance between *max_temp* and this point is larger than a pre-set value, and in the meanwhile, the amplitude of this point is higher than a pre-set value, then the corresponding frequency component at this point is considered as active. If the point satisfying the above conditions exists, it will imply that there are at least two active components in the current BLIMF (including *max_temp*). Obviously, this means mode-mixing has occurred. The pseudocode of this judgment algorithm is detailed in Algorithm 1. 

**Algorithm 1:** Pseudo-code of the proposed mode-mixing judgement method

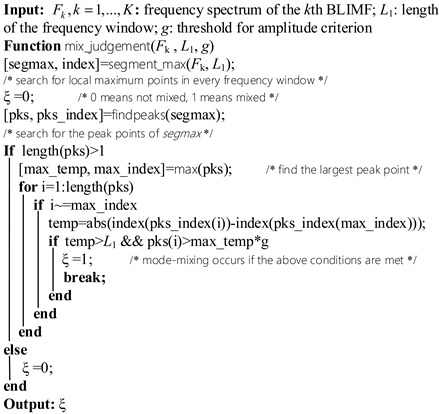



Follow the workflow in Figure 1 and the decision rule in Algorithm 1, the optimal mode number *K* can be obtained in just a few steps. 

### 3.2. Effective BLIMF Selection

Based on the AVMD algorithm presented in the previous section, the PD signal is decomposed into *K* BLIMFs, and each BLIMF contains only one active component. However, not all these active components are PD components. In fact, the generating mechanism of PD electromagnetic signal and its propagation path determine that it exhibits characteristics of damped oscillation and steep rising edge in the time domain, while narrowband periodic noise or white noise does not show such characteristic. Therefore, in this paper, BLIMF with following features is defined as effective BLIMF (abbreviate as eBLIMF): (i) with central frequency in UHF band (i.e., higher than 0.3GHz); (ii) exhibits damped oscillation in time domain; (iii) has steep rising edge. Based on the above analysis, the kurtosis operator which is sensitive to abrupt change is selected as the indicator, and the decision rule is formulated as:

(10) $$eBLIMF=u_{k}\;\;\;\;s.t.\;\;kurtosis\left(u_{k}\right)>\varepsilon_{1}\;\;and\;f_{0}(u_{k})>0.3GHz$$

where $\varepsilon_{1}$ is a pre-set threshold. *f*_0_( ) is the central frequency operator, and *kurtosis*( ) is the kurtosis operator, which is calculated as:

(11) $$kurtosis(X)=E{\left(x-\mu \right)}^{4}/\sigma^{4}=\frac{1}{n}\sum_{i=1}^{n}{\left(x_{i}-\bar{x}\right)}^{4}/{\left(\frac{1}{n}\sum_{i=1}^{n}{\left(x_{i}-\bar{x}\right)}^{2}\right)}^{2}$$

where X is a discrete signal defined in Section 2.2, and $\bar{x}$ denotes its mean value. 

### 3.3. SSA-based Shrinkage method

In this section, a denoising algorithm based on SSA and shrinkage method is designed. For each eBLIMF, the Hankel matrix is constructed and be decomposed into several sub-space matrices *A_i_* by SVD. Since there is only one dominant component in each eBLIMF, the grouping step described in Section 2.2 becomes easy. Assume the SVs are sorted in descending order, and the ratio of each SV to the sum of all SVs are denoted as $\left\{q_{1},q_{2},\ldots ,q_{R}\right\}$ (*R* is the rank of Hankel matrix), a common way to choose DSVs is to compute the cumulative sum of q until its value reached a proper threshold $\varepsilon_{2}$. This process can be expressed as:

(12) $$\sum_{i=1}^{N_{1}}q_{i}>\varepsilon_{2}$$

Therefore, the first $N_{1}$ SVs are decided as DSVs. For each DSV, its corresponding sub-space matrices *A_i_* is computed by Equation (9), then the reconstructed signal ${\hat{X}}_{i}$ based on *A_i_* can be obtained by the diagonal averaging method. In order to further suppress the white noise in ${\hat{X}}_{i}$, the shrinkage technology typically used in WT-based denoising is employed [9]. Specifically, the multiplicative threshold rescaling scheme with *sqtwolog* rule is adopted, which is expressed as:

(13) $$\zeta_{i}=\frac{MAD\left|{\hat{X}}_{i}\right|}{0.6745}\sqrt{2\log(n_{i})},\;\;\;i\in {\mathbb{R}}_{DSVs}$$

where $n_{i}$ is the length of ${\hat{X}}_{i}$, ${\mathbb{R}}_{DSVs}$ is the set of DSVs, $\zeta_{i}$ is the threshold of ${\hat{X}}_{i}$. Afterwards, the hard threshold function is applied to denoise the recovered signal ${\hat{X}}_{i}$, given by:

(14) $${\hat{x}}_{ij}=\left\{ \begin{array}{ll} {\hat{x}}_{ij}, & {\hat{x}}_{ij}>\zeta_{i},\;j=1,2,\ldots ,n_{i}\\ 0, & otherwise \end{array}\right.$$

where ${\hat{x}}_{ij}$ is the *j*th element of ${\hat{X}}_{i}$. At last, for all DSVs, the denoised ${\hat{x}}_{ij}$ are added to form the denoised eBLIMF: 

In Equation (15), ${\hat{\hat{X}}}_{j}$ is the *j*th element of the denoised eBLIMF, and $N_{1}$ has been explained in Equation (12). The whole procedure of SSA-based shrinkage method is illustrated in Algorithm 2.

(15) $${\hat{\hat{X}}}_{j}=\sum_{i=1}^{N_{1}}{\hat{x}}_{ij}$$

**Algorithm 2:** Pseudo-code of thr proposed SSA-based Shrinkage denoising method

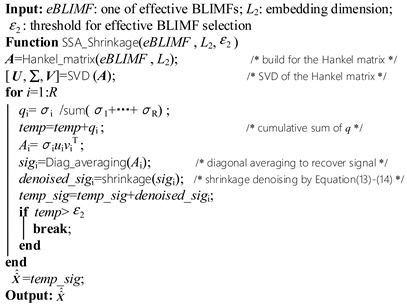



### 3.4. Implementation Procedure of Proposed AVMDSSA Method

**Figure 2:**
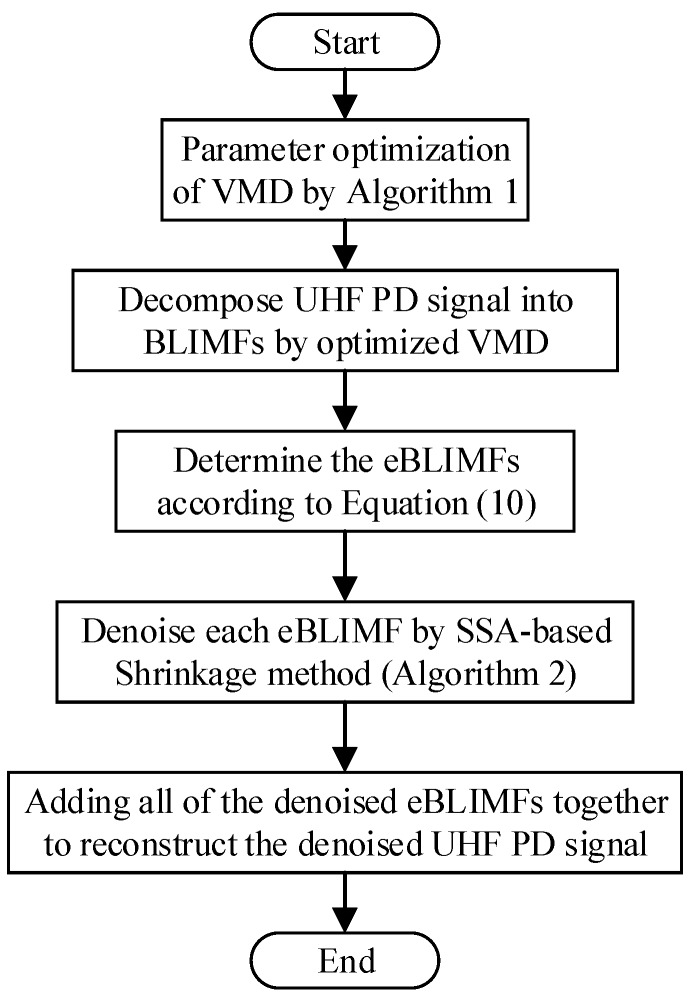
Schematic diagram of AVMDSSA denoising method.

Based on the above descriptions, the schematic diagram of AVMDSSA method is demonstrated in Figure 2, and the overall process is summarized as follows:

(i) Optimization of the number of modes *K* by gradually increasing its value and judging whether there is mode-mixing happened in each BLIMF at every step.
(ii) Decompose the UHF PD signal into a set of BLIMFs by VMD with the optimal *K* parameter, then a kurtosis-based selection method is employed to pick out the eBLIMFs.
(iii) For each eBLIMF, the SSA-based Shrinkage denoising method is applied to suppress the white noise, and summation of all denoised eBLIMFs will recover the denoised UHF PD signal.

## 4. Simulative Case Study

### 4.1. Synthetic UHF PD Signal

To verify the ability of the proposed method to deal with complex signal, a synthetic UHF PD signal with multiple PD components and various noises is simulated. Here, we use the Single Exponential Decay Oscillating (SEDO) pulse and Double Exponential Decay Oscillating (DEDO) pulse to simulate the PD signal. Their respective mathematical expressions are as follows [6]:

(16) $$S_{1}=Be^{-t/\tau_{1}}\sin\left(2\pi f_{c}t\right)$$

(17) $$S_{2}=B(e^{-t/\tau_{1}}-e^{-t/\tau_{2}})\sin\left(2\pi f_{c}t\right)$$

where *B* is the amplitude, $\tau_{1}$ and $\tau_{2}$ are attenuation coefficients, $f_{c}$ is the center frequency. The synthetic UHF PD signal combines one SEDO pulse, two DEDO pulses and white noise. In addition, two periodic narrowband noises (PNN) are added, which is formulated as:

(18) $$S_{narrowband}=B\sin\left(2\pi f_{c}t\right)$$

**Figure 3:**
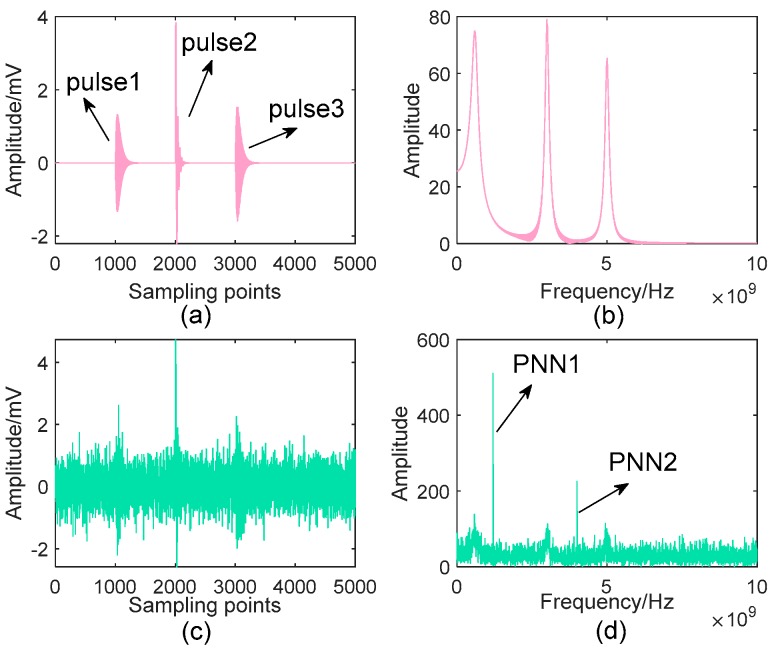
Synthetic UHF PD signal and its spectrum: (**a**) Pure signal; (**b**) Spectrum of the pure signal; (**c**) Noisy signal; (**d**) Spectrum of the noisy signal.

Detailed parameters of this simulative signal are listed in Table 1, and its Signal to Noise Ratio (SNR) is −5.576 dB. Figure 3 shows the time-domain waveforms and frequency spectra of the pure and noisy PD signal, respectively. One can see from Figure 3c that the original components are almost unrecognizable from the noisy signal. Here, the proposed denoising method is applied to suppress the complex noise, and some relevant parameters are shown in Table 2. 

### 4.2. Denoising Results

**Figure 4:**
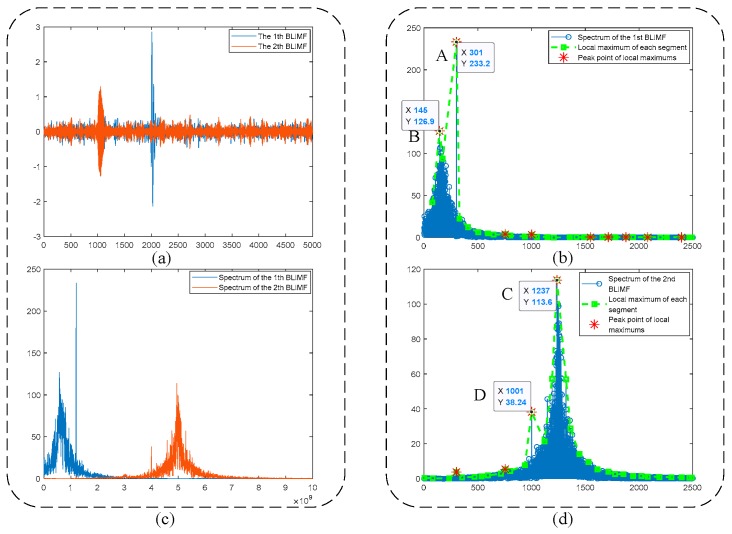
Optimization process when *k* = 2: (**a**) Time-domain waveforms of BLIMFs; (**b**) Local maximums of 1st BLIMF and its peak points; (**c**) Spectra of BLIMFs; (**d**) Local maximums of 2nd BLIMF and its peak points.

Starting with *K* = 2, the process for denoising of the PD signal shown in Figure 3c is depicted in Figure 4. It is observed from Figure 4b,d that the local maximums (dashed green line with square markers) and their peak points (red asterisks) can reflect well the active components in frequency spectrum. According to Algorithm 1 and the parameters listed in Table 2, Point A and Point B in Figure 4b are judged as belonging to two different components, respectively (criterion 1: 301−145 > 80 and criterion 2: 126.9 > 233.2 × 0.1 are satisfied simultaneously). Similarly, Point C and Point D in Figure 4d are decided to represent diverse components. The above results show that when *K* = 2, the mode-mixing occurs in the decomposed BLIMFs. In fact, when *K* = 2, the mode number of the VMD algorithm is much smaller than the number of the real components in original signal (i.e., 5), leading to the so-called *undersegmentation* phenomenon [18]. Therefore, according to the proposed algorithm, *K* should be increased. 

**Figure 5:**
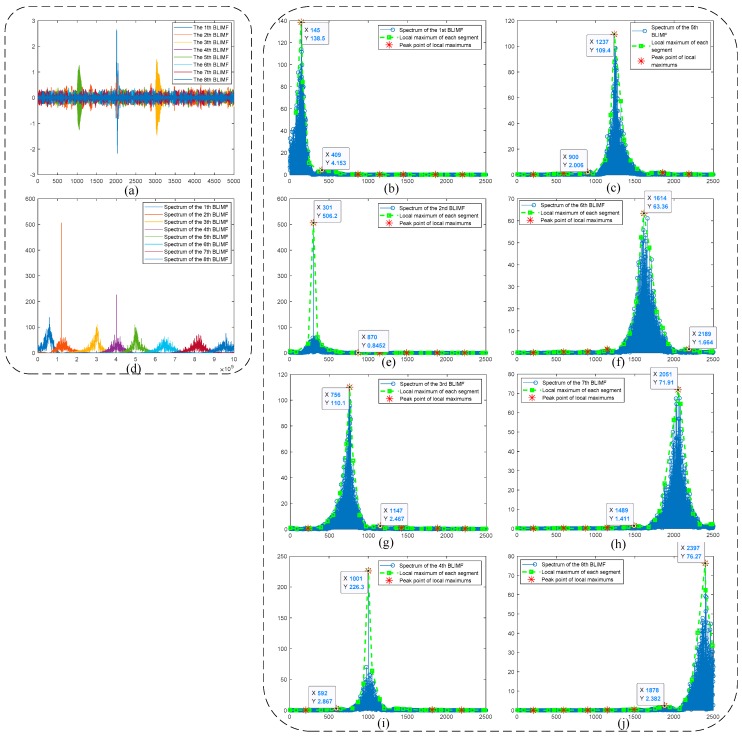
Optimization process when k = 8: (**a**) Time-domain waveforms of BLIMFs; (**b**,**c**) Local maximums of 1st and 5th BLIMFs and their peak points; (**d**) Spectra of BLIMFs; (**e**–**j**) Local maximums of 2nd, 6th, 3rd, 7th, 4th, 8th BLIMFs and their peak points.

For each *K* value, the mode-mixing judgement of BLIMF is carried out. Considering the limited space of our paper, we omit the graphic illustration of every optimization process, and provide only the judgement results in Table 3. As can be seen, when *K* increases to 8, there is no mode-mixing happened in all BLIMFs, indicating that the noisy signal has been decomposed completely. This can be seen from Figure 5, which shows the optimizing procedure when *K* = 8. Comparing Figure 5d with Figure 3d intuitively, one can see that all the active components in original signal are separated well. In addition, by using the decision rule in Algorithm 1, only one dominant component is identified in every BLIMF, that is, there is no mode-mixing in all decomposed modes. Other subgraphs of Figure 5 demonstrate these decision processes. 

**Figure 6:**
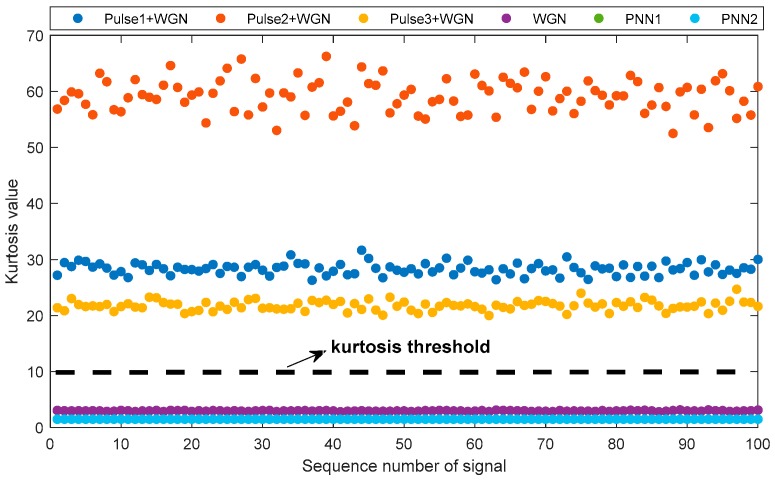
Kurtosis values for different types of signals.

The second step of AVMDSSA is to pick out the eBLIMFs. To obtain an appropriate kurtosis threshold, we simulate six different types of signals, and each type contains one hundred randomly generated signals. Details of these signal are given below: (1) WGN: the white gaussian noise (WGN) with power of 0 dBw; (2) Pulse1+WGN/ Pulse2+WGN/ Pulse3+WGN: PD signal Pulse1, Pulse2, Pulse3 added by WGN, respectively, and SNR = 0 dB; (3) PNN1/PNN2: the noise signal PNN1, PNN2 with arbitrary amplitude between 0.1 mV and 0.2 mV. The kurtosis results of all these signals are given in Figure 6. As can be readily seen, sequences containing PD signals have kurtosis values greater than those without PD signals. Particularly, kurtosis values of sequences containing PD signals are all larger than 10. Therefore, we choose this value as the threshold. It should be noted that when extremely severe noise is mixed in PD signal, the kurtosis value will decrease, thus this threshold should be reduced accordingly. However, considering that the SNR of BLIMF after VMD is not likely too low, the threshold 10 is suitable.

**Figure 7:**
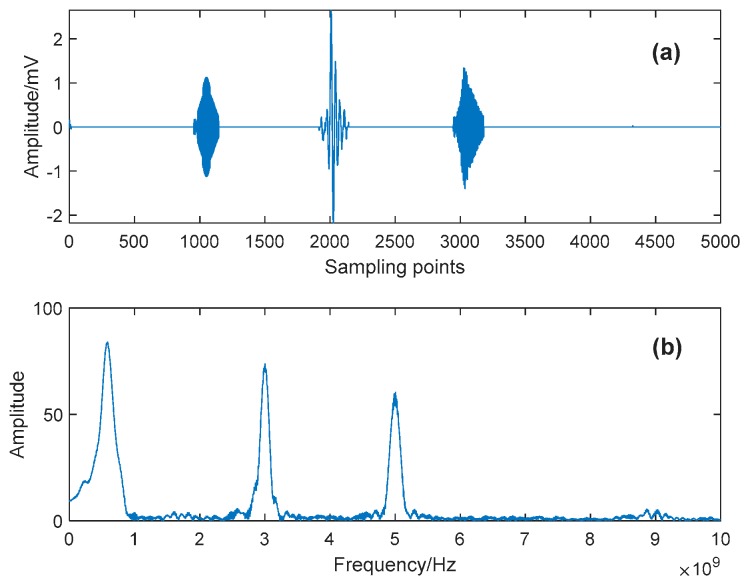
Denoising results by AVMDSSA: (**a**) Time-domain waveform; (**b**) Spectrum.

After obtaining the eBLIMFs, the SSA-based Shrinkage method is applied, and final results are depicted in Figure 7. Comparing with Figure 3, one can easily observe that the various noises in the noisy PD signal have been well suppressed. Moreover, those effective PD components in original signal have been completely reserved. This example shows the capability of the proposed method in not only the noise reduction, but also the feature preservation.

### 4.3. Noise Robustness

**Figure 8:**
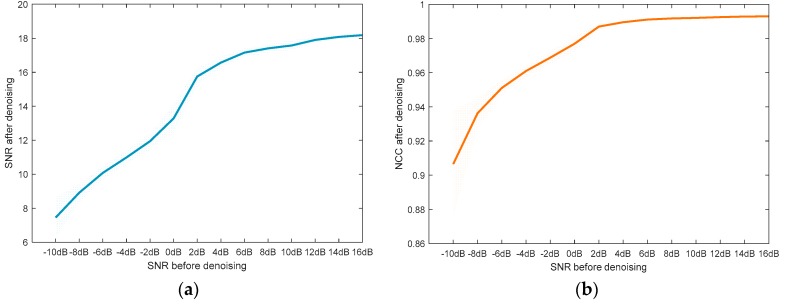
Evaluation of robustness of AVMDSSA: (**a**) SNR values after denoising; (**b**) NCC values after denoising. The bold solid line in each plot represents the mean value of corresponding index, and the semi-transparent regions are the value space of each index between its positive and negative standard error.

Before discussing the robustness of the proposed method, two common evaluation indices are introduced to evaluate the denoising performance, which are the normalized correlation coefficient (NCC) and SNR. Detailed calculating formulas of these indices can be found in [10,25]. In this subsection, noisy UHF PD signals with different SNR levels are generated by adding WGN to the pure signal shown in Figure 3a. One hundred signals are simulated in each SNR level, and the values of SNR are as follows: −10 dB, −8 dB, −6 dB, −4 dB, −2 dB, 0 dB, 2 dB, 4 dB, 6 dB, 8 dB, 10 dB, 12 dB, 14 dB, 16 dB. Denoising results of these signals by AVMDSSA are depicted in Figure 8.

As can be seen from Figure 8a, the SNR value has been greatly improved after denoising. Even when the SNR of raw signal drops to −10 dB, it can still be raised to about 7.46 dB after denoising. In addition, with the increasement of SNR of the raw signals, the SNR results of the denoised signals also improve drastically, and gradually converge to their mean value. Similar denoising performance can also be found in Figure 8b by another index NCC. Therefore, we can conclude that the AVMDSSA method can achieve satisfactory denoising performance under different noise level. In the meanwhile, we should admit that the denoising performance will fluctuate to some extent at low SNR scenario, but still within the acceptable range.

### 4.4. Comparison with Traditional Denoising Methods

**Figure 9:**
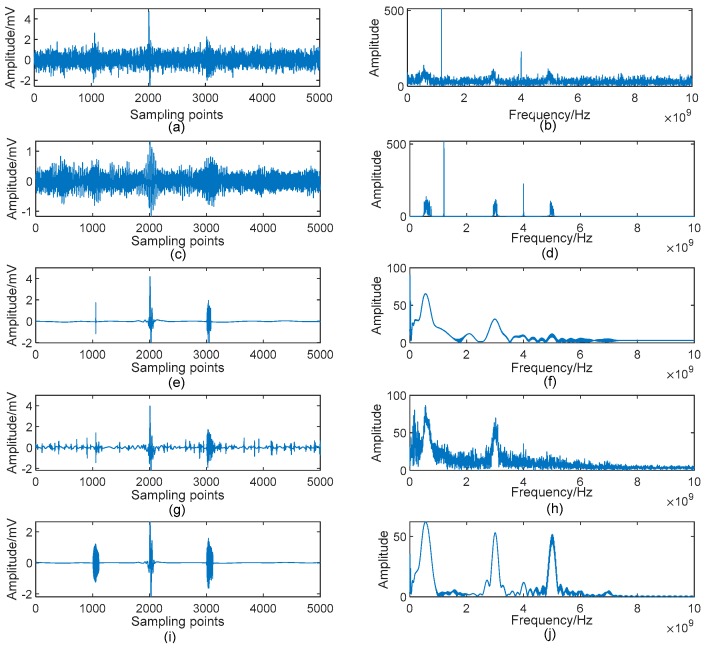
Denoising results by different algorithms: (**a**) Noisy signal; (**b**) Spectrum of the noisy signal; (**c**) Denoised signal by *Method* 1; (**d**) Spectrum of (c); (**e**) Denoised signal by *Method* 2; (**f**) Spectrum of (**e**); (**g**) Denoised signal by *Method* 3; (**h**) Spectrum of (**g**); (**i**) Denoised signal by *Method* 4; (**j**) Spectrum of (**i**).

In this part, four other denoising algorithms are employed to compare with the proposed method, which are as follows. Method 1: Adaptive Singular Value Decomposition (ASVD) [15]; Method 2: Wavelet Shrinkage [9]; Method 3: Complete Ensemble Empirical Mode Decomposition with Adaptive Noise (CEEMDAN) and Wavelet Threshold [26]; Method 4: Optimized VMD and Wavelet Transform [8]; Method 5: AVMDSSA. Denoising results of the signal shown in Figure 3c are given in Figure 9. 

Figure 9c,d show that the ASVD algorithm in [15] is unable to remove the narrowband noise of UHF PD signal. This is mainly because the grouping scheme of singular components in this method is energy-oriented, and the periodic narrowband noise is exactly energy-rich. Thus, ASVD is failed to denoise this kind of noise. From Figure 9e,h, one can easily see that both of Method 2 and Method 3 loss the 5 GHz component (i.e., Pulse 1). Meanwhile, both methods introduce strong low frequency interferences. In addition, there are many small oscillations in Figure 9g which called the Pseudo-Gibbs phenomenon, resulting in severe high-frequency interferences in its spectrum. In contrast, Method 4 shows better performance in preserving PD components. However, slight noise is still existed in its spectrum. Comparing these results with Figure 7, there is no doubt that the presented method AVMDSSA shows superior performance not only in noise suppression, but also in feature preservation. 

**Figure 10:**
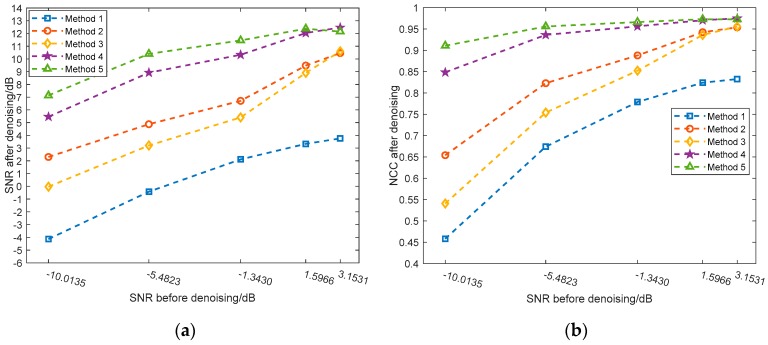
Quantitative results by different algorithm under various SNR levels: (**a**) SNR results; (**b**) NCC results.

To quantitatively evaluate the performance of the above algorithms, noisy UHF PD signals in five different SNR levels are simulated by adding WGN and PNN to the pure signal shown in Figure 3a. To obtain a more credible result, one hundred signals are generated for each SNR level, and the average results of SNR and NCC after denoising are depicted in Figure 10. One can readily observe that the AVMDSSA algorithm exhibits better denoising performance over other methods in either SNR or NCC, even in an extremely noisy circumstance.

## 5. Laboratorial Case Study

### 5.1. Laboratorial PD Measurement Setup

**Figure 11:**
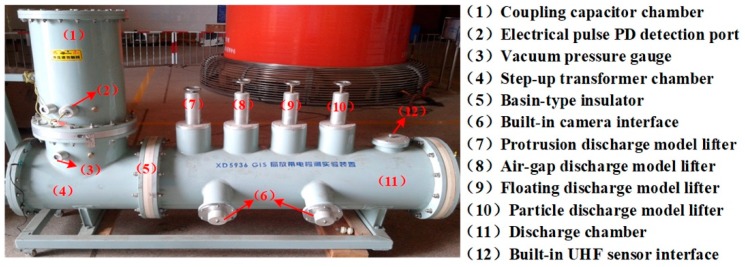
Simulative Gas Insulated Switchgear (GIS) chamber and its related accessories.

**Figure 12:**
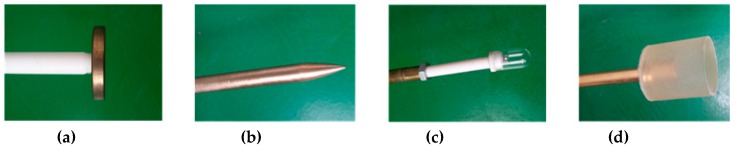
Artificial defect models used in our tests: (**a**) Floating discharge model; (**b**) Protrusion discharge model; (**c**) Particle discharge model; (**d**) Air-gap discharge model.

To obtain the measured UHF PD data under different insulation defects, PD experiments were carried out in the high voltage laboratory of the China Electric Power Research Institute. Experimental setup and necessary descriptions of its accessories are given in Figure 11. Additionally, Figure 12 shows the physical drawings of all artificial defect models. During the test, the PD signals were coupled by the built-in UHF sensor firstly, then they were sampled and stored by a high-speed digital storage oscilloscope (LeCroy WaveRunner 204Xi-A, 10GS/s, 2GHz).

**Figure 13:**
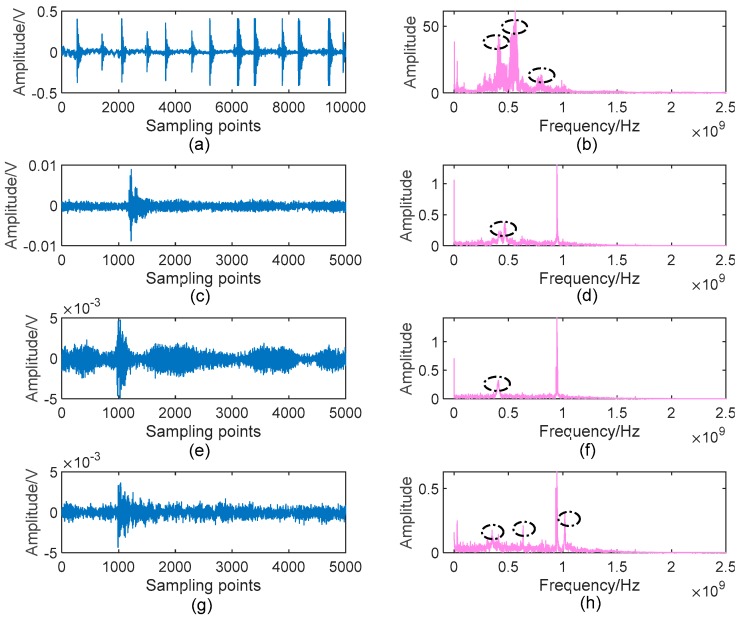
Measured UHF PD signals by laboratorial setup: (**a**) Typical waveform of Type 1; (**b**) Spectrum of (**a**); (**c**) Typical waveform of Type 2; (**d**) Spectrum of (**c**); (**e**) Typical waveform of Type 3; (**f**) Spectrum of (**e**); (**g**) Typical waveform of Type 4; (**h**) Spectrum of (**g**). Possible PD components in each spectrum are marked with black dash-dotted ellipse.

In our experiments, all defects were tested, and we denote the UHF PD signals from the floating discharge model, protrusion discharge model, particle discharge model and air-gap discharge model as Type1, Type 2, Type 3 and Type 4, respectively. Typical waveforms of these discharge signals are shown in Figure 13. As can be seen, these PD signals are polluted by different noise. Furthermore, one can observe that there are multiple PD components in Figure 13b,h, making the denoising process more difficult. 

### 5.2. New Evaluation Indices for Practical Situation

Considering that the pure PD signal is not available in practice, it is impossible to calculate the evaluation index such as SNR or NCC. In addition, existing indicators only focus on the degree of noise reduction, while ignoring the preservation of the signal features after denoising. To give a more comprehensive evaluation of the denoising algorithm under practical conditions, two new indices are designed in this paper:

(i) *Index* 1: The ratio of the Shannon entropy of the signal before and after denoising

As we all know, the measured PD signals in practice are usually mixed with randomly distributed, uncertain noise. This uncertainty will decrease as the noise is suppressed, and the more thoroughly the noise is removed, the less the uncertainty will be. Therefore, the Shannon entropy, a measure of uncertainty, is employed to quantify this kind of uncertainty. The designed *Index* 1 can be calculated by the following formula:

(19) $$Index1=\frac{entropy\left(\left|nor\left(denoised\_sig\right)\right|\right)}{entropy\left(\left|nor\left(noisy\_sig\right)\right|\right)}$$

where *denoised_sig* and *noisy_sig* denote the UHF PD signal after and before denoising, respectively. *nor*( ) and *entropy*( ) are the operators of normalization and Shannon entropy. Obviously, lower *Index* 1 implies better denoising performance

(ii) *Index* 2: The ratio of the summation of eBLIMFs’ maximum frequency values before and after denoising

Another key issue of denoising is the feature preservation. The basic idea behind this index is that the more the features are retained after denoising, the less the spectrum amplitude of effective components will decrease. Therefore, we adopt the criterion in Section 3.2 to decide the eBLIMFs of *noisy_sig* and *denoised_sig*, expressed as *eBLIMFs*_1_ and *eBLIMFs*_2_ respectively. Then, the maximum frequency values of each eBLIMF in *eBLIMFs*_1_ are added to form the *MF*_1_. Following the same way, we can calculate the other parameter *MF*_2_. Then, the *Index* 2 is obtained by the ratio of *MF*_2_ to *MF*_1_. This process can be formulated as follows:

(20) $$Index2=\frac{\sum_{i\in eBLIMF_{2}}max\left(\left|fft\left(eBLIMF_{i}\right)\right|\right)}{\sum_{j\in eBLIMF_{1}}max\left(\left|fft\left(eBLIMF_{j}\right)\right|\right)}$$

where *fft*( ) is the Fast Fourier Transform (FFT) operator. And clearly, a larger *Index* 2 means the superior performance of the denoising algorithm in feature preservation.

### 5.3. Denoising Results and Comparisons

**Figure 14:**
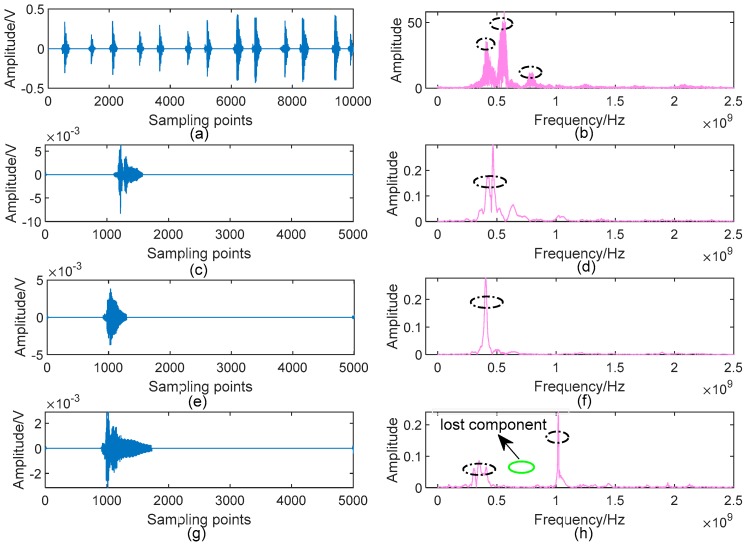
Denoised UHF PD signals by AVMDSSA: (**a**) Denoised signal of Type 1; (**b**) Spectrum of (**a**); (**c**) Denoised signal of Type 2; (**d**) Spectrum of (**c**); (**e**) Denoised signal of Type 3; (**f**) Spectrum of (**e**); (**g**) Denoised signal of Type 4; (**h**) Spectrum of (**g**). Detected PD components in each spectrum are marked with black dash-dotted ellipse, and the lost components in (**h**) is marked by green solid ellipse.

Figure 14 gives the denoising results of the UHF PD signals shown in Figure 13 by using the proposed AVMDSSA algorithm. Comparing with Figure 13, one can see that the low-frequency noise and communication carrier noise in each raw signal are suppressed completely, especially for Type 1 and Type 4, in which multiple PD components are existed. More importantly, all possible PD components marked in Figure 13 are well retained in denoised signals.

**Figure 15:**
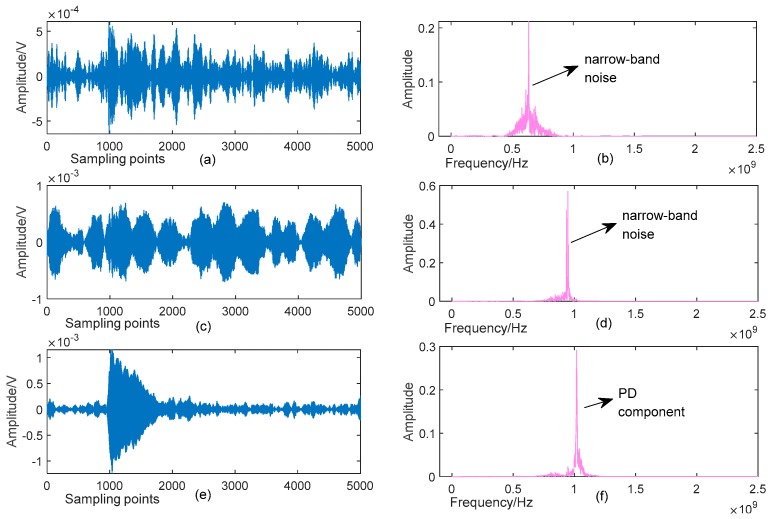
Some decomposed BLIMFs of *Type* 4 signal: (**a**) The 4th BLIMF; (**b**) Spectrum of (**a**); (**c**) The 5th BLIMF; (**d**) Spectrum of (**c**); (**e**) The 6th BLIMF; (**f**) Spectrum of (**e**).

One may have noticed that one possible PD component in Figure 13h is lost in Figure 14h. In fact, it is easy to observe from the decomposed BLIMFs shown in Figure 15 (only plot the 4th, 5th and 6th BLIMF for example) that the missed component (i.e., the 4th BLIMF) is one kind of narrowband noise, rather a PD component. On the contrary, the time-domain waveform of the frequency component in Figure 15f shows obvious characteristics of PD (according to the decision criteria of eBLIMF discussed in Section 3.2), thus it can be identified as a real PD component. The above results demonstrate the effectiveness of our method in noise suppression and feature preservation for different kinds of measured UHF PD data.

To compare the AVMDSSA with traditional methods quantitatively, the evaluation indices are computed by Equations (19) and (20). For each PD type, one hundred signals are used as the input, and the average results of the two indices are listed in Table 4. It can be observed that Method 1 has the worst denoising results, indicating that the ASVD method may not be suitable for processing complex UHF PD signals. In contrast, the AVMDSSA method shows the best results in either *Index*1 or *Index2*, showing that it has superior performance over traditional methods in both noise suppression and feature retention. In addition, it should be noted that although Method 1 has the largest *Index2* among all methods, it does not mean that it has the best feature retention capability. In fact, the main reason for this is that it fails to remove the noise very well, causing a relatively smaller decrease of the amplitude than other methods.

## 6. Field Case Study

**Figure 16:**
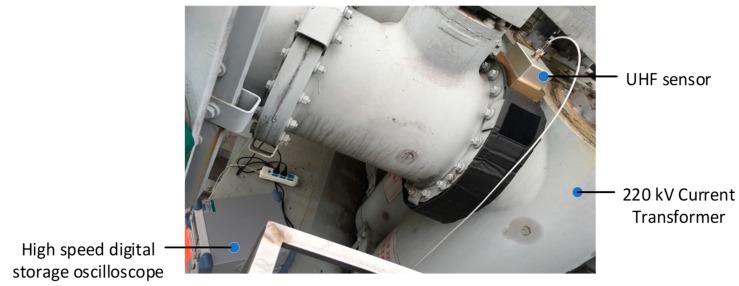
Field picture of the PD detection.

**Figure 17:**
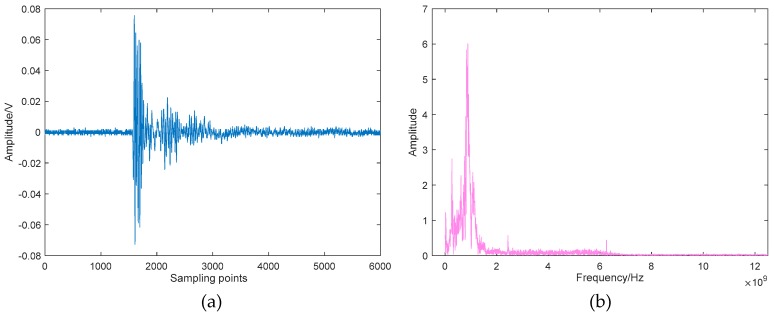
UHF PD signal measured in field test: (**a**) Time-domain waveform; (**b**) Spectrum.

In this section, the field UHF PD data from a 220 kV current transformer are analyzed. The suspected PD signal radiates outward through the resin sprue on the basin insulator, and then be coupled by the UHF sensor and sent to the oscilloscope (25 GS/s, 6GHz) for sampling and storage. The picture of the field test is given in Figure 16, and the typical time-domain waveform and its spectrum are depicted in Figure 17. As can be seen, although the metal shell of GIS can shield most of the external interferences, the captured signal still has a complex composition, which makes the denoising work more challenging.

**Figure 18:**
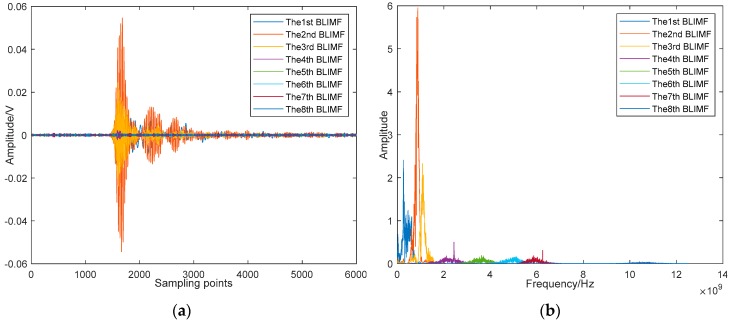
BLIMFs decomposed by VMD using the optimal *K*: (**a**) Time-domain waveforms; (**b**) Spectra.

**Figure 19:**
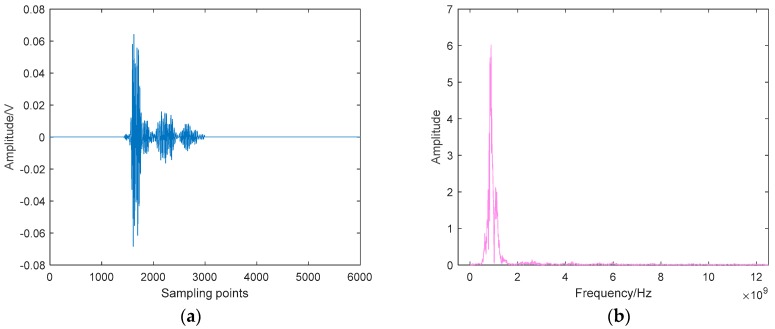
Final denoising result by AVMDSSA: (**a**) Time-domain waveform; (**b**) Spectrum.

By using the proposed method, the mode number of VMD is optimized first, and the optimal value of *K* is 8 in this example. Figure 18 shows the decomposed BLIMFs of the above signal by applying the optimal VMD. A comparison between Figure 17b and Figure 18b shows that there are no active frequency components mixing or missing, which means the decomposition is complete. Next, the eBLIMFs will be decided according to Equation (10) by calculating their central frequencies and kurtosis values. Results of this step are listed in Table 5, in which can we see that only BLIMF2 and BLIMF3 are judged as eBLIMFs. At last, the SSA-based Shrinkage scheme is used to further remove the white noise in each eBLIMF. The final denoising result is shown in Figure 19.

Again, we use the newly designed indices to evaluate the performance of AVMDSSA under this field situation. The average computation results of fifty measured signals are as follow: *Index*1: 0.4860; *Index*2: 0.9723. This result demonstrates that the AVMDSSA method can effectively reduce the uncertainty of the measured data. And what’s more, there is almost no loss of the characteristic components after the denoising process, which is critical to the subsequent processing and diagnosis.

## 7. Discussion

In this paper, a novel denoising method namely AVMDSSA is developed. To comprehensively evaluate the performance of AVMDSSA, three case studies are conducted, and we discuss the results as follows:

(i) Simulative case

In the simulative case study, complex UHF PD signals with heavy noise are simulated. One can easily see from Figure 4b,d that if the decomposition by VMD is incomplete (i.e., the value of *K* is smaller than the actual number of active components in the raw signal), there will be multiple local maximum points in the BLIMF. On the contrary, every decomposed BLIMF should contain only one active component if *K* is equal to the number of actual components, as can be seen from Figure 5. The optimization process by the proposed AVMD algorithm is given in Table 3. After the selection of eBLIMFs and an SSA-based shrinkage algorithm, the final denoising result is obtained. Comparing Figure 7 with Figure 9, one can readily observe the superiority of AVMDSSA over other methods. In addition, the results of the noise robustness tests (depicted in Figure 8) and quantitative comparison (depicted in Figure 10) are all demonstrate the effectiveness of our method.

(ii) Laboratorial case

To examine the performance of AVMDSSA by real UHF PD signal, four kinds of laboratorial PD data are collected. In addition, we define two new evaluation indices for denoising method from the perspective of noise suppression and feature retention, respectively. The effectiveness of the proposed AVMDSSA method is readily shown in Figure 14, even for the signal that contains multiple PD components. By using the newly defined indices for each method, we can conclude from Table 4 that AVMDSSA shows the best performance (lowest *Index*1, highest *Index*2).

(iii) Field Case

The UHF PD signals from a real high-voltage current transformer are also employed in this paper, and the denoising results shown in Figure 19 are satisfactory, suggesting that our method has the application potential in practical scenarios.

The above discussions prove the effectiveness of the presented method. Moreover, it is worth mentioning that all the denoising results of AVMDSSA are obtained by using the same parameters listed in Table 2, which indicates the robustness of this method.

Compared with our previous denoising study in reference [8], contributions in this work are summarized as follows: (i) the parameter optimization method adopted in this paper has more explicit physical meaning and faster searching speed; (ii) the SSA-based shrinkage method developed in this work shows better denoising performance compared with the Wavelet Shrinkage method used in [8]; (iii) more comprehensive assessment of the denoising method is given in this study, including the noise robustness test, newly designed evaluation indices, and the field test. All these advancements show that the current method achieves much improvement compared to our previous work.

Despite all the advantages, we should admit that the efficiency of AVMDSSA needs to be improved. For example, the average time consumption of AVMDSSA for a signal with length of 5000 is 3.6 s (hardware: Intel Core i5 CPU, 16 GB RAM; software: MATLAB 2018b), while this value can rise to more than 10 s when dealing with a signal with length of 10000. Such high computational cost mainly comes from the SVD algorithm used in SSA. To a certain extent, this restricts the real-time application of our method in embedded hardware. However, for offline applications that are not very sensitive to computational complexity, the proposed method still shows great advantages.

To suppress the electromagnetic interference in PD signals more thoroughly, improvements in hardware design are also important. For example, a reliable metal shell for the UHF sensor will prevent the external noise from coupling into the detection system to a large extent. Besides, considering that the optical fiber is a kind of ideal transmission medium due to its advantages such as high sensitivity, immunity to electromagnetic interference and stability in harsh environments, it can be used in the data transmission module of the PD detection system. In summary, only when the noise suppression measures are taken into account in both of hardware and software design, can the PD detection system play a more stable and reliable role in practice.

## 8. Conclusions

A key challenge for PD detection and diagnosis is how to suppress the complicated interfering noise. In this paper, a novel denoising method for UHF PD signal is proposed. First, the mode number of VMD is automatically decided by a mode-mixing judgement algorithm. Next, those decomposed BLIMFs which contain PD components are selected based on their central frequency and kurtosis values, namely eBLIMFs. Finally, the residual white noise in each eBLIMF is further reduced by SSA-based Shrinkage method, and the denoised PD signal is obtained by adding all these denoised eBLIMFs together. The proposed AVMDSSA method is applied to three cases, and the following conclusions are obtained:

(i) The mode-mixing decision rule proposed in this paper works very well in all cases, enabling the AVMDSSA method to quickly determine the appropriate *K* value.
(ii) In the simulative case, a complex synthetic UHF PD which contains three PD pulses and two kinds of noises is employed to examine our method. The results show that the proposed method can reduce all kinds of noises to a large extent, and in the meanwhile, all PD components are well retained. In addition, the results of robustness testing and comparison demonstrate its reliability and superiority.
(iii) For the measured data, two new evaluation indices are presented by considering both of the capabilities of noise suppression and feature preservation. By using these newly designed indices, the effectiveness of AVMDSSA in laboratory experiments and field tests are identified.

Our future work will focus on improving the efficiency of the denoising method, making it more valuable in practical application.

## Figures and Tables

**Table 1:** Parameters of the synthetic UHF PD signal.

Items	Type	Amplitude: *B*, mV	Attenuation Coefficients: τ_1_, τ_2_, ns	Center Frequency: *f*_c_, GHz	Sampling Rate: *f_s_*, GHz
Pulse1	DEDO	5	1.2, 2.5	5	20
Pulse2	SEDO	5	1.5, --	0.6	20
Pulse3	DEDO	6	1.2, 2.5	3	20
PNN1	PNN	0.2	--, --	1.2	20
PNN2	PNN	0.1	--, --	4	20

**Table 2:** Parameters of AVMDSSA.

Parameters	*L*_1_	*L*_2_	*ε*_1_	*ε*_2_	*g*
Description	Length of each segment used in adaptive VMD	The embedding dimension for Hankel matrix construction	Kurtosis threshold for eBLIMF selection	Threshold for dominant singular values	Used for mode-mixing judgement
Value	80	100	10	0.95	0.1

**Table 3:** Mode-mixing judgement results of BLIMFs for each *K* value. ‘1′ means mode-mixing happened, while ‘0′ means no mode-mixing happened.

*K* Value	1st BLIMF	2nd BLIMF	3rd BLIMF	4th BLIMF	5th BLIMF	6th BLIMF	7th BLIMF	8th BLIMF
2	1	1	—	—	—	—	—	—
3	1	1	1	—	—	—	—	—
4	1	1	1	0	—	—	—	—
5	1	1	1	0	0	—	—	—
6	0	0	1	1	0	0	—	—
7	0	0	1	1	0	0	0	—
8	0	0	0	0	0	0	0	0

**Table 4:** Average results of *Index*1 and *Index*2 for different situations.

Method	*Method* 1		*Method* 2		*Method* 3		*Method* 4		*Method* 5	
	*Index*1	*Index*2	*Index*1	*Index*2	*Index*1	*Index*2	*Index*1	*Index*2	*Index*1	*Index*2
*Type*1	0.7713	0.9113	0.6740	0.5620	0.6850	0.5106	0.6801	0.6901	0.4490	0.7490
*Type*2	0.8200	0.8600	0.6250	0.6204	0.3380	0.7380	0.1075	0.7575	0.0889	0.8189
*Type*3	0.7653	0.8200	0.5336	0.6336	0.3132	0.7532	0.0715	0.7315	0.0804	0.8014
*Type*4	0.9153	0.9198	0.4106	0.5106	0.5518	0.6118	0.0763	0.6815	0.0728	0.7828

**Table 5:** Central frequencies and kurtosis values of the BLIMFs of the above UHF PD signal.

Indicators	BLIMF1	BLIMF2	BLIMF3	BLIMF4	BLIMF5	BLIMF6	BLIMF7	BLIMF8
Central frequency (GHz)	0.236	0.879	1.075	2.433	3.621	5.117	6.250	10.41
Kurtosis	22.36	38.69	37.95	5.94	3.14	2.93	3.19	2.72
